# Activation of NLRP3 Inflammasome by Advanced Glycation End Products Promotes Pancreatic Islet Damage

**DOI:** 10.1155/2017/9692546

**Published:** 2017-11-05

**Authors:** Xiang Kong, Ai-Ling Lu, Xin-Ming Yao, Qiang Hua, Xiao-Yong Li, Li Qin, Hong-Mei Zhang, Guang-Xun Meng, Qing Su

**Affiliations:** ^1^Department of Endocrinology, Xinhua Hospital, Shanghai Jiaotong University School of Medicine, Shanghai 200092, China; ^2^Department of Endocrinology, The First Affiliated Hospital of Wannan Medical College, Yijishan Hospital, Wuhu 241001, China; ^3^Engineering Technology Research Center of Polysaccharides Drug, Wuhu, Anhui Province 241002, China; ^4^Key Laboratory of Molecular Virology & Immunology, Institut Pasteur of Shanghai, Shanghai Institutes for Biological Sciences, Chinese Academy of Sciences, Shanghai 200031, China

## Abstract

Accumulation of advanced glycation end products (AGEs) contributes to ageing and age-related diseases, especially type 2 diabetes. The NLRP3 inflammasome, as a vital component of the innate immune system, is implicated in the pathogenesis of type 2 diabetes. However, the role of the NLRP3 inflammasome in AGE-induced pancreatic islet damage remains largely unclear. Results showed that administration of AGEs (120 mg/kg for 6 weeks) in C57BL/6J mice induced an abnormal response to glucose (as measured by glucose tolerance and insulin release), pancreatic *β*-cell ultrastructural lesion, and cell death. These effects were associated with an excessive superoxide anion level, significant increased protein expression levels for NADPH oxidase 2 (NOX2), thioredoxin-interacting protein (TXNIP), NLRP3, and cleaved IL-1*β*, enhanced caspase-1 activity, and a significant increase in the levels of TXNIP–NLRP3 protein interaction. Ablation of the NLRP3 inflammasome or treatment with antioxidant *N*-acetyl-cysteine (NAC) clearly ameliorated these effects. In conclusion, our results reveal a possible mechanism for AGE-induced pancreatic islet damage upon NLRP3 inflammasome activation.

## 1. Introduction

Advanced glycation end products (AGEs) are generated nonenzymatically, and the formation of AGEs is greatly accelerated by prolonged hyperglycemia in patients with diabetes mellitus (DM) [1, 2]. AGE accumulation is one of the main factors that contribute to ageing and is an important element of etiopathology of age-related diseases, especially type 2 DM and its complications [3–5]. Recent studies have pointed out that AGE accumulation directly causes insulin-producing *β*-cell dysfunction and apoptosis *in vivo* [6–9]. These effects are attributed, at least in part, to an increase in cellular reactive oxygen species (ROS) production. Nonetheless, the precise role of AGE-mediated ROS release in *β*-cell damage remains unclear.

Inflammation is a critical mechanism leading to *β*-cell dysfunction and death, wherein the inflammatory cytokines play an important role [10, 11]. Among the various proinflammatory cytokines, interleukin-1*β* (IL-1*β*) plays a major role in DM. Clinical studies reported that inhibition of IL-1*β* by either IL-1 receptor antagonist or IL-1*β* antibody in patients with type 2 DM leads to improvement in glycemia and *β*-cell function [12, 13]. It is known that IL-1*β* secretion is predominantly mediated by the cysteine protease caspase-1, which is mainly activated by inflammasomes, especially the nucleotide-binding domain leucine-rich repeat containing receptor, the pyrin domain-containing 3 (NLRP3) inflammasome [14].

Thioredoxin-interacting protein (TXNIP), a member of the arrestin protein superfamily, regulates diabetic islet *β*-cell apoptosis and dysfunction [15–17]. TXNIP is the endogenous inhibitor and regulator of thioredoxin, which maintains the balance of both the cellular antioxidant and antiapoptotic system [18]. Recent research demonstrates that the dissociation of TXNIP from thioredoxin in a ROS-sensitive manner could activate the NLRP3 inflammasome and induce IL-1*β* release, namely, oxidative stress links TXNIP to inflammasome activation [19–21].

Increasing evidence suggests that metabolic stress (glucose, free fatty acids, and human amyloid polypeptide) appears to activate the IL-1 system through the NLRP3 inflammasome in pancreatic islet [19, 22, 23]. However, it remains unclear whether the NLRP3 inflammasome participates in the AGE-induced *β*-cell damage, although the latter process is companied by ROS production. As a result, in the current study, we test the hypothesis that the ROS/TXNIP pathway contributes to AGE-induced activation of NLRP3 inflammasome, which results in the release of active IL-1*β*, leading to pancreatic islet *β*-cell damage *in vivo*.

## 2. Materials and Methods

### 2.1. Animals and Reagents

Ten-week-old male NLRP3 knockout (NLRP3 KO) mice were used in our study as we described previously [24]. Age-matched C57BL/6J mice were obtained from the Jackson Laboratory and used as the wild-type (WT) control group. All of the mice were maintained in specific pathogen-free facilities at the Experimental Animal Centre of Xinhua Hospital. All studies were approved by the Institutional Animal Care and Use Committee (IACUC) at the Xinhua Hospital, Shanghai Jiaotong University School of Medicine.

All of the reagents were purchased from Sigma unless stated otherwise. Monoclonal mouse anti-NLRP3 antibody was obtained from Adipogen Inc. Polyclonal rabbit anti-TXNIP and insulin antibodies were purchased from Abcam Inc. Polyclonal rabbit anti-glucagon, NADPH oxidase 2 (NOX2), apoptotic speck-like protein (ASC), and IL-1*β* antibodies were purchased from Santa Cruz Inc. Dihydroethidium (DHE) probes, caspase-1 activity, and terminal deoxynucleotidyl transferase dUTP nick end labeling (TUNEL) assay kits were purchased from Beyotime Biotechnology Inc. IL-1*β* ELISA kit was purchased from R&D Systems. Mouse insulin ELISA kit was purchased from Shibayagi Inc.

### 2.2. Preparation of AGEs

The AGEs used in this study were described previously [8, 9] and prepared as in Makita et al. [25]. In brief, bovine serum albumin (BSA, 50 mg/mL) was incubated under sterile condition with glyceraldehyde (0.1 M) in phosphate buffer (pH 7.4, 0.2 M) for seven days. The unincorporated sugar was removed by dialysis. Nonglycated BSA, incubated in the absence of glyceraldehyde, was used as a negative control. The AGE preparation was tested for the presence of endotoxins using a limulus amebocyte lysate (LAL) reagent yielding an endotoxin level of less than 15 EU/L.

### 2.3. Laboratory Rodent Studies

In the first part of the experiment, mice were separated into the following groups (*n* = 10 each group): WT mice with BSA, NLRP3 KO mice with BSA, WT mice with AGEs, and NLRP3 KO mice with AGEs. BSA (used as a control) or AGEs were daily administered intraperitoneally at the dosage of 120 mg/kg of body weight for 6 weeks according to our previous report [9].

In the second part of this study, groups of C57BL/6J mice (*n* = 6 each group) were given daily intraperitoneal injections of either AGEs or BSA for 6 weeks as mentioned above. An additional subgroup of mice injected with AGEs (*n* = 6) received treatment with the antioxidant *N*-acetyl-cysteine (NAC, 40 mM in drinking water), at a concentration that provided sufficient *in vivo* antioxidant capacity [26, 27]. We did not include a group of C57BL/6J mice treated with NAC alone in this study because NAC does not influence the NLRP3 inflammasome activity in unstimulated human peripheral blood monocytes [28].

### 2.4. Intraperitoneal Glucose Tolerance Test (GTT), Insulin Releasing Test (IRT), and Intraperitoneal Insulin Tolerance Test (ITT)

Overnight-fasted mice were intraperitoneally injected with a 10% glucose solution (1.5 mg/g body weight). For intraperitoneal glucose tolerance test (GTT), glucose levels were determined at different time points with a glucometer. For insulin releasing test (IRT), blood was collected at different time points after glucose loading, and insulin levels were determined with an ELISA kit.

For intraperitoneal insulin tolerance test (ITT), mice were fasted 6 hours and then intraperitoneally injected with human regular insulin (0.75 U/kg). Glucose levels were measured at 0, 15, 30, 45, and 60 min with a glucometer.

### 2.5. Determination of Physiological Indices

At the end of the present study, overnight-fasted mice were anaesthetized with an intraperitoneal injection of sodium pentobarbital. Blood samples were collected, centrifuged to obtain serum, and kept at −80°C until assayed. Fasting blood glucose levels were determined by the glucose oxidase method. Fasting insulin concentrations were measured with an ELISA kit.

### 2.6. Immunofluorescent Staining

A portion of the pancreas was fixed in 4% paraformaldehyde, embedded in paraffin, and cut into 5 *μ*m sections. For TUNEL staining, the pancreatic sections were incubated with TUNEL reagent for 1 h in the dark. Nuclear staining was achieved by incubating with DAPI. In addition, a positive control was prepared from permeabilized pancreatic sections preincubated with deoxyribonuclease 1 to induce DNA strand breaks. For immunofluorescent staining, fixed pancreatic sections were heated for 15 min in boiling 10 mM citrate buffer (pH = 6.0) for antigen retrieval. Then, sections were probed with anti-insulin (1 : 200), glucagon (1 : 150), or IL-1*β* (1 : 100) antibodies, followed by incubation with specific secondary antibodies. Negative controls were prepared, in which the antibody probing of the pancreatic sections was substituted by PBS buffer addition, at the same concentration of nonimmune rabbit immunoglobulin G. Sections were photographed by fluorescent microscopy and analyzed using ImageJ software as described in our previous reports [9, 29].

### 2.7. Detection of Superoxide Anion in Mice Pancreatic Islet

As in our previous report [9], a portion of the pancreas was embedded in O.C.T. embedding medium. Sections (10 *μ*m) were incubated for 30 minutes with DHE (10 *μ*M) to evaluate pancreatic superoxide anion levels in situ. DHE is oxidized by superoxide anion to yield ethidium, which is trapped intracellularly by intercalation into the DNA. Ethidium fluorescence was quantified using ImageJ software [9].

### 2.8. Transmission Electron Microscopy

Pancreatic tissues from the tail of the pancreas were harvested and fixated in 2.5% glutaraldehyde. Thereafter, fixed samples were treated with 1% osmium tetroxide, dehydrated, and embedded. After localization of islets under a light microscope, the tissues were cut into ultrathin sections. The sections were put on uranyl acetate and lead citrate before examined in a transmission electron microscope.

### 2.9. Caspase-1 Activity Assay

The caspase-1 activity of pancreatic tissue lysates was measured using a colourimetric assay. This assay is based on the ability of caspase-1 to change acetyl-Tyr-Val-Ala-Asp p-nitroaniline (Ac-YVAD-pNA) into the yellow formazan product p-nitroaniline (pNA). Production of pNA per minute in tested samples was used as a measure of the level of caspase-1 activity and inflammasome activation. Results are expressed as fold increase in caspase-1 activity.

### 2.10. Western Blot Analysis and Immunoprecipitation

Equal protein of pancreatic tissue lysates (40 *μ*g) was separated by electrophoresis on a sodium dodecyl sulphate polyacrylamide gel (SDS-PAGE). Separated proteins were transferred electrophoretically to PVDF membranes and then incubated with the primary antibodies against NLRP3 (1 : 500), ASC (1 : 800), IL-1*β* (1 : 200), TXNIP (1 : 500), and tubulin (1 : 1000) overnight and with the correspondent secondary peroxidase-conjugated anti-rabbit or mouse antibodies. Immunoprecipitation (IP) was performed as described previously. Total protein (100 mg) was immunoprecipitated with anti-TXNIP antibody (5 *μ*g/mL) and incubated with A/G agarose beads overnight. Precipitated proteins were analysed by SDS-PAGE and blotted with primary antibodies (anti-TXNIP and anti-NLRP3). Antibody-bound proteins were detected with an enhanced chemiluminescence (ECL) kit (Millipore). Blots were quantified by densitometry using Image J software. The intensity of the bands was normalized to that of tubulin or TXNIP.

### 2.11. Statistical Analysis

Data were expressed as the mean ± standard deviation (S.D.). The differences among groups were determined by the use of one-way analysis of variance followed by Newman–Keuls test. A *p* value of less than 0.05 was considered to be statistically significant.

## 3. Results

### 3.1. NLRP3 Knockout Improves the Abnormal Response to Glucose in Mice Administration of AGEs

**Figure 1:**
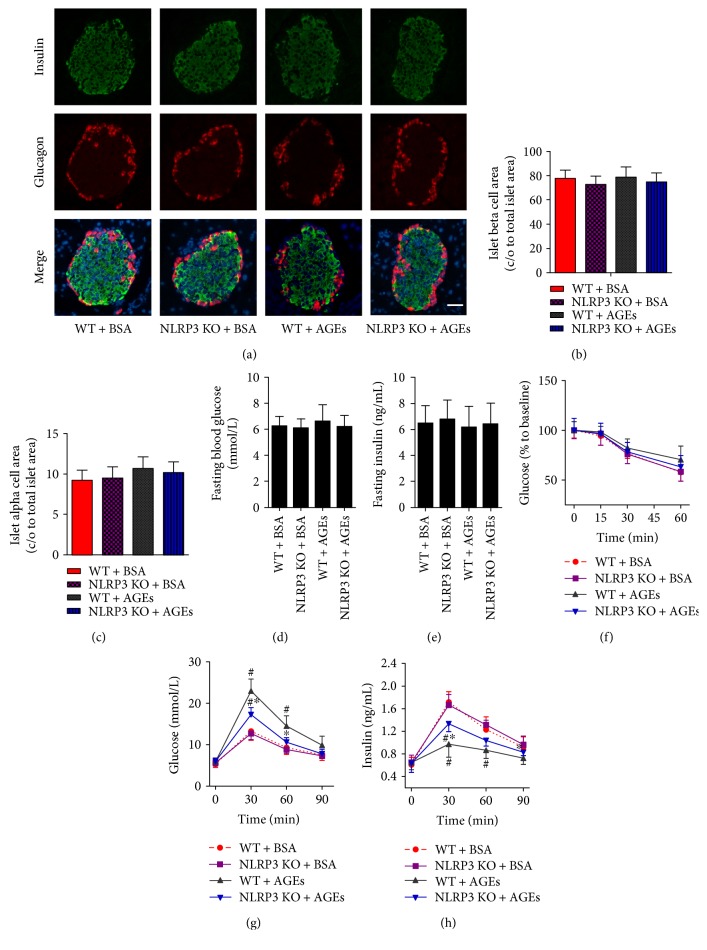
NLRP3 knockout improves the abnormal response to glucose in mice administration of AGEs. C57BL/6J (WT) and NLRP3 knockout (NLRP3 KO) mice were injected intraperitoneally with 120 mg/kg AGEs or BSA for 6 weeks. (a) Pancreatic sections were stained by insulin (green), glucagon (red), and DAPI (blue), with representative islets shown (scale bar = 50 *μ*m). Histogram represents quantitative analysis of insulin-positive beta cell area (b) and glucagon-positive alpha cell area (c) in each experimental group. *n* = 5 − 6 per group. Fasting glucose (d) and insulin (e) as well as insulin tolerance (f) were not obviously changed in each group. GTT (d) and IRT (e) were performed after intraperitoneal injection of glucose (1.5 mg/g). *n* = 5 per group. Ablation of NLRP3 improved the abnormal glucose metabolism induced by AGE injection. Values are expressed as mean ± SD, ^#^*P* < 0.05 versus WT + BSA group. ^∗^*P* < 0.05 versus WT + AGEs group.

NLRP3 KO mice were used to understand the role of the NLRP3 inflammasome in AGE-induced pancreatic islet damage. WT and NLRP3 KO mice were injected intraperitoneally with AGEs or BSA (control) daily for 6 weeks. We first performed insulin and glucagon double immunofluorescence staining of pancreatic sections for insulin and glucagon to evaluate the islet morphology in mice. As shown in Figure 1(a), the 6-week AGE treatment caused few morphological changes in the pancreatic islet. Total *β*-cell and *α*-cell mass were not significantly different among the four groups (Figures 1(b) and 1(c)).

There were no significant differences in fasting blood glucose level (Figure 1(d)) and insulin concentration (Figure 1(e)) among the four groups. Whole-body insulin tolerance, as assessed by the intraperitoneal ITT, was not significantly different among groups (Figure 1(f)). Then, an intraperitoneal GTT was performed to assess metabolic alterations, namely, glucose tolerance and glucose-stimulated insulin release. Blood glucose level after glucose loading was significantly raised in WT mice treated with AGEs compared with WT mice treated with BSA (Figure 1(g)), which correlated with a lower insulin level in AGE-treated WT mice as compared to BSA-treated WT mice (Figure 1(h)). These effects were not owed to the development of insulin resistance because the AGE treatment did not affect fasting glucose and insulin levels or whole-body insulin sensitivity, which compared to those of BSA-treated WT mice. Therefore, these results demonstrate that administration of AGEs directly impaired *β*-cell function *in vivo*. Interestingly, AGE-dependent effects such as higher level of blood glucose and impaired insulin secretion after glucose loading were significantly ameliorated by the ablation of NLRP3 (Figures 1(g) and 1(h)).

### 3.2. NLRP3 Knockout Decreases Islet *β*-Cell Apoptosis in Mice Administration of AGEs

**Figure 2:**
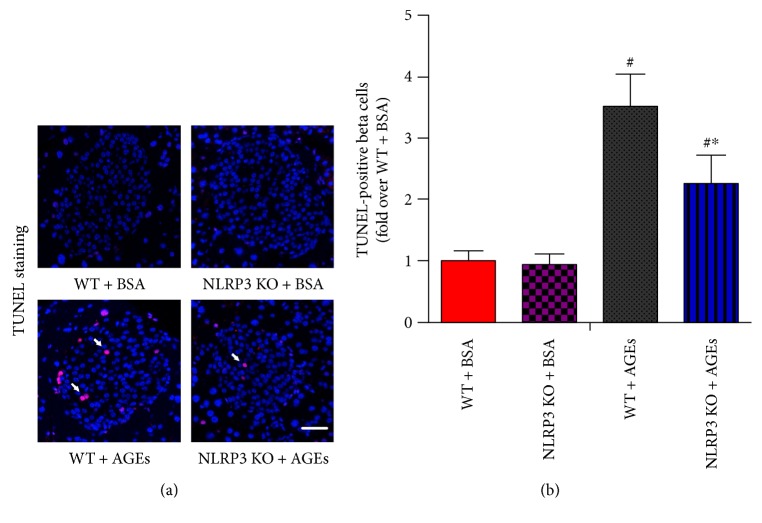
NLRP3 knockout decreases islet *β*-cell apoptosis in mice administration of AGEs. WT and NLRP3 KO mice were injected intraperitoneally with 120 mg/kg AGEs or BSA for 6 weeks. (a) Representative photomicrographs of TUNEL-positive cells in mice pancreatic sections. Scale bar = 50 *μ*m. (b) Histogram represents quantitative analysis of TUNEL-positive *β*-cells per islet in each experimental group. *n* = 5 per group. Values are expressed as mean ± SD, ^#^*P* < 0.05 versus WT + BSA group. ^∗^*P* < 0.05 versus WT + AGEs group.

Previous studies have clarified that impaired *in vivo* insulin secretion can result from a decrease in *β*-cell survival or function or a combination of both [30]. As shown in Figure 2, the number of apoptotic cells identified by positive TUNEL staining was much higher in AGE-treated WT mice compared to the number of apoptotic cells in AGE-treated NLRP3 KO mice. These data suggest that deletion of NLRP3 gene protects islet *β*-cell from AGE-induced death *in vivo*.

### 3.3. NLRP3 Knockout Reverts Ultrastructural Lesion of Islet *β*-Cell in Mice Administration of AGEs

**Figure 3:**
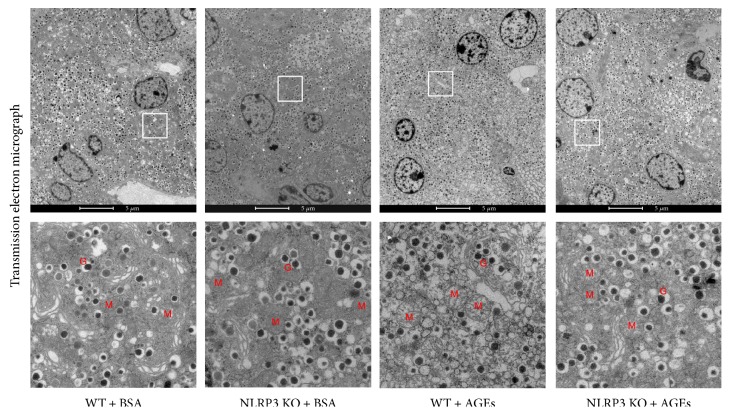
Ultrastructural changes in WT and NLRP3 KO mice after administrated with AGEs. The insulin-secretory granules were almost unaltered in each group. The swollen mitochondria in AGE-treated WT mice were protected by the NLRP3 ablation. M: mitochondria; G: granules.

We used transmission electron microscopy to better characterize the protective effect of the NLRP3 deletion at single cell level. As shown in Figure 3, the electron micrograph of *β*-cells within islet displayed the swollen mitochondria in AGE-treated WT mice compared with those in BSA-treated WT mice, although the insulin-secretory granules were almost unaltered among groups. Ablation of NLRP3 led to a significant amelioration of AGE-induced mitochondrial damage in the *β*-cells within islets.

### 3.4. NLRP3 Inflammasome Activation Is Involved in AGE-Induced Mice Pancreatic Islet Damage

**Figure 4:**
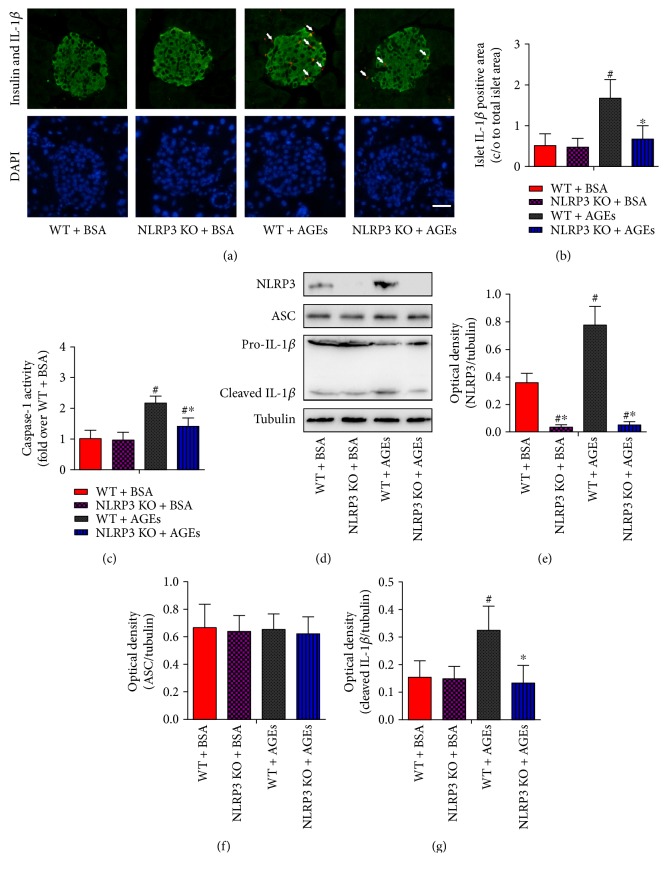
NLRP3 inflammasome activation is involved in AGE-induced mice pancreatic islet damage. WT and NLRP3 KO mice were injected intraperitoneally with 120 mg/kg AGEs or BSA for 6 weeks. (a) Pancreatic sections were stained by insulin (green), IL-1*β* (red), and DAPI (blue), with representative islets shown (scale bar = 50 *μ*m). (b) Histogram represents quantitative analysis of IL-1*β*-positive cell area in each experimental group. *n* = 5 per group. (c) The caspase-1 activity in pancreas tissue lysates was assessed using Ac-YVAD-pNA. *n* = 5 − 6 per group. (d) Immunoblotting analysis was performed for NLRP3, ASC, and active IL-1*β* (p17) in pancreas tissue lysates of mice. Histograms represent optical density values of NLRP3 (e), ASC (f), and active IL-1*β* (g) normalized to the corresponding tubulin. *n* = 3 − 4 per group. Values are expressed as mean ± SD, ^#^*P* < 0.05 versus WT + BSA group. ^∗^*P* < 0.05 versus WT + AGEs group.

To directly address the involvement of the NLRP3 inflammasome in AGE-induced islet lesions, we investigated the local processing of IL-1*β*, which is the ultimate step of inflammasome activation. Immunofluorescence results suggested that, although IL-1*β* expression was relatively low in normal WT mice as consistent with a previous report [31], IL-1*β* staining could still be detected in the islets. Nevertheless, daily AGE injection induced a significant elevated immunoexpression level of IL-1*β* in WT mice, and this change was reversed by the NLRP3 deletion (Figures 4(a) and 4(b)).

Since the immunostaining of IL-1*β* reflects the total IL-1*β* protein expression including precursor and mature forms, we performed immunoblotting to analyze IL-1*β* partitioning into the two forms. Caspase-1 assays were also performed to detect the enzymatic activity of caspase-1. As shown in Figures 4(c), 4(d), 4(e), 4(f), and 4(g), AGEs caused a substantial increase in the expression of NLRP3 protein, activation of caspase-1, and maturation of IL-1*β* in WT mice, whereas ASC expression levels remained constant. Deletion of NLRP3 prevented the occurrence of AGE-induced effects of enhanced caspase-1 activity and increased activation of IL-1*β* (17 kDa subunit) (Figures 4(c) and 4(d)).

### 3.5. Effects of NAC on AGE-Induced Mice Pancreatic NLRP3 Inflammasome Activation

**Figure 5:**
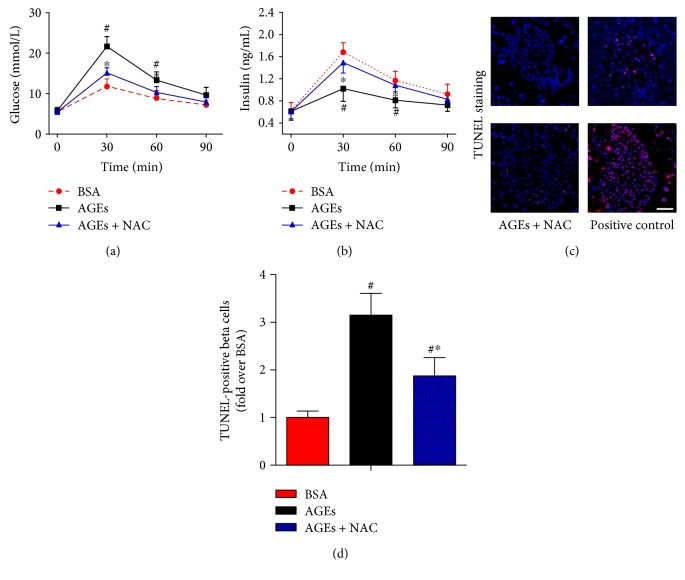
Effects of NAC treatment on the abnormal response to glucose and islet *β*-cell apoptosis in mice administration of AGEs. C57BL/6J mice were injected intraperitoneally with BSA, AGEs, or AGEs plus the antioxidant NAC (40 mM in drinking water) for 6 weeks. GTT (a) and IRT (b) were performed after intraperitoneal injection of glucose (1.5 mg/g). *n* = 6 per group. (c) Representative photomicrographs of TUNEL-positive cells in mice pancreatic sections. The positive control includes permeabilization of sections with deoxyribonuclease 1 to induce DNA strand breaks. Scale bar = 50 *μ*m. (d) Histogram represents quantitative analysis of TUNEL-positive *β* cells per islet in each experimental group. *n* = 6 per group. Values are expressed as mean ± SD, ^#^*P* < 0.05 versus BSA group. ^∗^*P* < 0.05 versus AGEs group.

**Figure 6:**
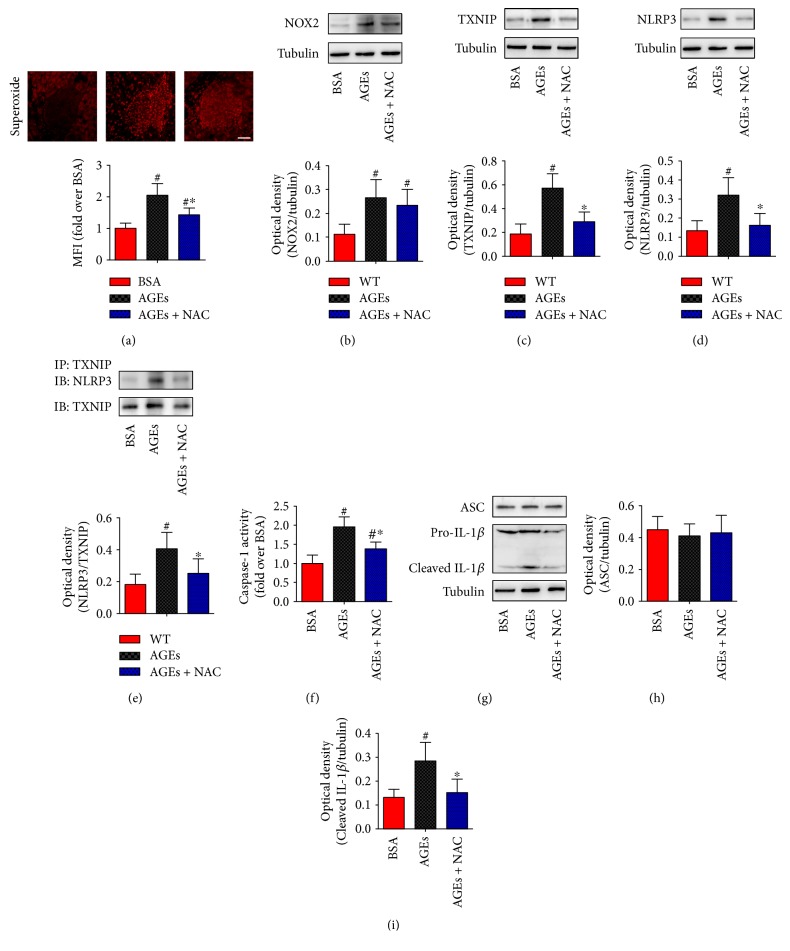
AGE-induced mice pancreatic NLRP3 inflammasome activation through ROS/TXNIP pathway. C57BL/6J mice were injected intraperitoneally with BSA, AGEs, or AGEs plus the antioxidant NAC (40 mM in drinking water) for 6 weeks. (a) Representative photomicrographs of mice pancreatic sections stained with DHE. Scale bar = 50 *μ*m. Histogram represents quantitative analysis of superoxide anion generation in each experimental group. *n* = 6 per group. Representative blots and Western blot analyses of NOX2 (b), TXNIP (c), and NLRP3 (d) protein expression in mice pancreas tissue lysates. *n* = 3 − 4 per group. (e) Representative blot and quantification of immunoprecipitation (IP) with TXNIP and blotting (IB) with NLRP3 showed higher association of TXNIP with NLRP3 (*n* = 3 per group), which was associated with increased caspase-1 activity ((f), *n* = 6 per group) and enhanced cleaved IL-1*β* expression ((g, h, i), *n* = 3 per group). Values are expressed as mean ± SD, ^#^*P* < 0.05 versus BSA group. ^∗^*P* < 0.05 versus AGEs group. MFI: mean fluorescence intensity.

We next explored whether the ROS/TXNIP pathway contributes to AGE-induced NLRP3 inflammasome activation. To this end, C57BL/6J mice were administered daily with BSA, AGEs, or AGEs plus NAC (a well-known ROS inhibitor) for 6 weeks. Our data illustrated that NAC treatment significantly improved islet *β*-cell function (Figures 5(a) and 5(b)) and inhibited *β*-cell death (Figures 5(c) and 5(d)). As shown in Figures 6(a) and 6(b), the superoxide anion production and NOX2 protein level were obviously enhanced in AGE-treated mice. These effects were associated with the significantly increased protein expression levels of TXNIP, NLRP3, and cleaved IL-1*β*, the enhanced caspase-1 activity, and an obvious increase in TXNIP-NLRP3 protein interaction, as assessed by coimmunoprecipitation (Figure 6(e)). Moreover, treatment with NAC significantly reduced the level of superoxide anion, inhibited the AGE-induced expression level of TXNIP, NLRP3, and cleaved IL-1*β*, decreased the caspase-1 activity, and reversed the phenotype for increased protein interaction levels between TXNIP and NLRP3 (Figure 6).

## 4. Discussion

Among the various AGE subtypes, the glyceraldehyde-derived AGEs (the predominant components of toxic AGEs) have been shown to play an important role in the development of inflammation and angiopathy in patients with DM [32–34]. In preliminary experiments, glyceraldehyde or glucose-derived AGEs were daily administered intraperitoneally to C57BL/6J mice at the dosage of 120 mg/kg of body weight for 6 weeks. Exposure to glyceraldehyde-derived AGEs caused significantly impaired glucose tolerance in C57BL/6J mice, which was not observed in mice treated with glucose-derived AGEs (data not shown). Therefore, the glyceraldehyde-derived AGEs were selected for subsequent studies and referred to as AGEs.

Proinflammatory cytokine overproduction is widely known to lead to DM. Several evidences suggest that the local IL-1*β* generation in pancreatic islets causes *β*-cell death and impairs their ability to produce insulin [35, 36]. There is growing support for the concept that AGEs play a pathological role due to their induction of proinflammatory cytokines, such as IL-1*β*, tumor necrosis factor-*α*, and so on [37–40]. AGEs directly increase IL-1*β* secretion without lipopolysaccharide priming in human peritoneal macrophages [40] and in adipose tissue of women with gestational diabetes [41]. The NLRP3 inflammasome appears as the sentinel, sensing metabolic stress and alarming the immune defense in pancreatic islets. NLRP3 activation leads to recruitment of the adaptor protein ASC and the effector protein caspase-1 to form the NLRP3 inflammasome complex, which ultimately is responsible for the cleavage and secretion of IL-1*β* [14]. Here, we showed that the AGE-induced increase in the maturation of pancreatic IL-1*β* was dependent on the NLRP3 inflammasome activation. Knockout of NLRP3 ameliorated the abnormal response to glucose (glucose tolerance and insulin release) in mice injected with AGEs. In addition, deletion of NLRP3 inflammasome protected the pancreatic *β*-cells from ultrastructural lesion and cell death caused by long-term AGE administration. Together, our results indicated that NLRP3 inflammasome activation is a key mechanism that participates in AGE-induced pancreatic damage. High concentrations of AGEs may trigger the NLRP3 inflammasome complex and result in the activation IL-1*β*. Subsequently, secreted IL-1*β* in the microenvironment exacerbates the chronic inflammatory response in pancreatic islets [42].

In line with our results, AGEs were reported to upregulate protein expression levels of the pattern recognition receptor for AGE (RAGE), increase ROS production, stimulate the activation of the NLRP3 inflammasome, and spark the development of renal injury in mice [43]. Treatment with RAGE antagonist obviously inhibits AGE/RAGE-induced ROS production and attenuates NLRP3 activation, consequently reducing the levels of IL-1*β* and attenuating abnormal kidney function in mice [43]. In addition, AGE treatment directly activates the NLRP3 inflammasome and stimulates mature IL-1*β* secretion in human placental tissues [44]. AGEs could also induce an inflammatory response in nucleus pulposus cells in a NLRP3 inflammasome-dependent manner related to the RAGE/NF-*κ*B pathway [45]. Other endogenous non-AGE ligands of RAGE, including S100A8 and S100A9, have been revealed to activate the NLRP3 inflammasome by stimulating the production of ROS [28]. Nevertheless, Kang et al. recently demonstrated that RAGE activates the melanoma 2 (but not NLRP3) inflammasome in acute pancreatitis [46]. A possible involvement of RAGE/NLRP3 inflammasome cannot be completely ruled out because macrophages used in their experiment were stimulated with histone and DNA, but not with AGEs. In fact, the components of the inflammasome, ASC, caspase-1, and NLRP3, are required for the development of inflammation in acute pancreatitis [47–49]. Knockout of NLRP3 gene protects against experimental acute pancreatitis [47] and chronic obesity-induced pancreatic damage [30]. Contrary to the results mentioned above, a recently published abstract has reported that pretreatment with AGEs attenuates diverse NLRP3 stimuli-triggered caspase-1 activation and IL-1*β* secretion in bone marrow-derived macrophages [50]. The direct effect of AGEs on immune cells requires further study.

Interestingly, little colocalization was observed between IL-1*β* and the *β*-cell marker insulin in the present study (Figure 4(a)), which suggests that islet resident and/or infiltrating macrophages may be the major source of proinflammatory cytokine in islets *in vivo*. NLRP3 inflammasome-mediated IL-1*β* production from infiltrating macrophages within the pancreas can contribute to the death of pancreatic *β*-cell and subsequent diabetes [51]. Further investigation to clarify the key cell types involved in AGE-stimulated IL-1*β* secretion in pancreatic islets is necessary.

Remarkably, ROS/TXNIP pathway has an essential role in triggering NLRP3 inflammasome activation and IL-1*β* secretion [19–21]. Both *in vivo* animal studies and *in vitro* cell culture studies have clarified that NADPH oxidase-related excessive ROS induces the separation of thioredoxin and TXNIP, resulting in the activation of NLRP3 inflammasome [42, 52, 53]. In the present study, we found that the administration of AGEs significantly increased the superoxide anion level through upregulation of NOX2 protein, which in turn contributed to an increase in expression levels of TXNIP and NLRP3 inflammasome components and an increase in protein interaction levels between TXNIP and NLRP3. Furthermore, treatment with NAC decreased the oxidative stress as indicated by a decrease in superoxide anion, led to the inhibition of the subsequent TXNIP-NLRP3 protein interaction levels and IL-1*β* secretion, and attenuated pancreatic islet injury. These results suggested that the NLRP3 expression and inflammasome activation in response to AGE stimulation are associated with ROS/TXNIP pathway *in vivo*.

## 5. Conclusion

Our research demonstrates that NLRP3 inflammasome activation is a key signalling mechanism in AGE-induced pancreatic islet damage. This study also provides direct *in vivo* evidence that NLRP3-deficient mice have suppressed pancreatic islet inflammatory response and damage upon AGE challenge. Thus, our work suggests that regulating the NLRP3 signalling might help control AGE-induced pancreatic islet damage in diabetic patients.
